# Memory-Based Mismatch Response to Frequency Changes in Rats

**DOI:** 10.1371/journal.pone.0024208

**Published:** 2011-09-06

**Authors:** Piia Astikainen, Gabor Stefanics, Miriam Nokia, Arto Lipponen, Fengyu Cong, Markku Penttonen, Timo Ruusuvirta

**Affiliations:** 1 Department of Psychology, University of Jyväskylä, Jyväskylä, Finland; 2 Institute for Psychology, Hungarian Academy of Sciences, Budapest, Hungary; 3 Institute for Empirical research in Economics, University of Zürich, Zürich, Switzerland; 4 Department of Neurobiology, A.I. Virtanen Institute, University of Eastern Finland, Kuopio, Finland; 5 Department of Mathematical Information Technology, University of Jyväskylä, Jyväskylä, Finland; 6 Department of Psychology, Turku Institute for Advanced Studies, University of Turku, Turku, Finland; Claremont Colleges, United States of America

## Abstract

Any occasional changes in the acoustic environment are of potential importance for survival. In humans, the preattentive detection of such changes generates the mismatch negativity (MMN) component of event-related brain potentials. MMN is elicited to rare changes (‘deviants’) in a series of otherwise regularly repeating stimuli (‘standards’). Deviant stimuli are detected on the basis of a neural comparison process between the input from the current stimulus and the sensory memory trace of the standard stimuli. It is, however, unclear to what extent animals show a similar comparison process in response to auditory changes. To resolve this issue, epidural potentials were recorded above the primary auditory cortex of urethane-anesthetized rats. In an oddball condition, tone frequency was used to differentiate deviants interspersed randomly among a standard tone. Mismatch responses were observed at 60–100 ms after stimulus onset for frequency increases of 5% and 12.5% but not for similarly descending deviants. The response diminished when the silent inter-stimulus interval was increased from 375 ms to 600 ms for +5% deviants and from 600 ms to 1000 ms for +12.5% deviants. In comparison to the oddball condition the response also diminished in a control condition in which no repetitive standards were presented (equiprobable condition). These findings suggest that the rat mismatch response is similar to the human MMN and indicate that anesthetized rats provide a valuable model for studies of central auditory processing.

## Introduction

Any sudden changes in the perceptual environment may signal a potential threat or opportunity. The rapid and automatic detection of such changes is therefore important. In humans, the preattentive detection of auditory changes is reflected by a mismatch negativity (MMN) component of event-related potentials [1]. MMN can be observed in response to rare tones (deviants) interspersed among frequent tones (standards) at about 100–250 ms from stimulus onset [2].

MMN is usually interpreted to reflect a comparison process in which a difference is detected between the current input and the representation of the standards in auditory sensory memory (memory-comparison hypothesis, [2]–[4]). Consistently, MMN is generally observed when silent inter-stimulus intervals (ISIs) (2–10 s, [5]–[7]) roughly corresponding to the length of the auditory sensory memory (1.5–4 s, [8], [9]) are used.

However, according to so-called refractoriness hypothesis, no comparison process between the input from the deviant stimulus and the memory trace of the standards is needed. Instead, it might be that standard stimuli induce refractoriness or adaptation in afferent pathways they repeatedly activate, so that deviant stimuli, merely by activating a distinct and hence “fresh” set of such pathways, only elicit an enhanced cortical N1 component instead of a genuine MMN component ([2], [4]; see also [10]). The memory-comparison hypothesis cannot, therefore, be accepted until the refractoriness hypothesis has been ruled out. This has been achieved by testing whether the MMN can be elicited without the standards in the series. The removal of standards has been performed in two alternate ways. Either the standards have been completely omitted, leaving only ‘control’ stimuli in the series (deviant-alone condition) or in a more controlled fashion by replacing standards with heterogeneous stimuli (with respect to the feature that differentiates deviants from standards) all presented with equal probability (equiprobable condition, see e.g. ref. [11] for frequency changes). An important advantage in this equiprobable condition is that it maintains the same overall presentation rate of the stimuli as in the oddball condition. These control procedures have consistently resulted in more negative responses to oddball-deviants than to control stimuli, indicating that a genuine memory-based MMN (e.g. [11]–[13]) and its analogy in infants (e.g. [14]) can be dissociated from refractoriness effects.

In animals, higher amplitude responses to deviants than standards (in some cases of *positive* polarity and hence termed ‘mismatch response’ here) have been reported several times (e.g. [15]–[24]). However, there are also reports of negative findings in rats [25]–[26]. Furthermore, when testing the refractoriness hypothesis, animal studies have relied solely on the deviant-alone condition [20], [23], [26]–[29], except one study reporting hippocampal mismatch responses in rabbits [30].

Here we study whether the mismatch response to frequency changes in urethane-anesthetized rats reflects the operation of a genuine memory-based comparison process by applying oddball and equiprobable conditions (modified from ref. [11]) to disentangle the mismatch response from possible refractoriness effects. To investigate the temporal span of the auditory sensory memory supporting the change detection, a previously unexplored issue in rats, different inter-stimulus intervals (ISIs) were applied. Across two animal groups, we used two different ISIs and two different deviant-standard frequency separations (standard constantly 4000 Hz, deviants 3800 Hz and 4200 Hz, ISIs 375 ms and 600 ms in one group and deviants 3500 Hz and 4500 Hz, ISIs 600 ms and 1000 ms in the other group). Based on prior studies of mismatch negativity in humans (e.g. [5], [6]) as well as mismatch potential in rabbits [15] we expected larger amplitude difference between standard and deviant responses for the shorter than the longer ISIs (375 ms vs. 600 ms and 600 ms vs. 1000 ms, in group 1 and group 2, respectively). We also expected that the larger deviant-standard frequency separation would better allow elicitation of the differential response than the smaller separation (5% vs. 12.5%) since similar findings exist in humans (e.g. [31]).

## Methods

### Subjects and surgery

Two groups of adult male Spraque Dawley rats were used in the study. Group 1 consisted of twenty animals, weighing 435–765 g, and group 2 consisted of thirteen animals, weighing 305–370 g. The animals were housed in metal cages, kept under a 12-h light-dark cycle and fed ad libitum. The experiments were approved by the Finnish National Animal Experiment Board (Permit code: ESLH-2007-00662), and carried out in accordance with the European Communities Council Directive (86/609/EEC) regarding the care and use of animals for experimental procedures. The presentation of the experimental blocks was counterbalanced between the animals. After the experiments, the anesthetized animal was immediately killed by cervical dislocation.

The animals were anesthetized with intraperitoneal injections of urethane (1.2 g/kg dose, 0.24 g/ml concentration, Sigma-Aldrich, St. Louis, MO, USA). Supplemental doses were injected if the required level of anaesthesia was not obtained. The level of anaesthesia was monitored by testing the withdrawal reflexes. The head of the animal was attached to the stereotaxic instrument (David Kopf Instruments, Model 962, Tujunga, CA, USA) using 45 degree ear bars. Under local anaesthesia (lidocaine 20%, Orion Pharma, Espoo, Finland) the skin and underlying muscles were removed and a unilateral craniotomy was performed to expose a 4×4 mm region of dura over the auditory cortex in the left hemisphere (coordinates for the recording area: 4.5–6.5 mm posterior and 3–5 mm ventral to bregma). The tip of a Teflon-insulated stainless steel wire (200 µm in diameter, A-M Systems, Chantilly, VA) was positioned on the surface of the dura on the basis of on-line recorded epidural potentials to tone stimuli that were similar to those later used in the actual experiment. Two stainless steel skull screws (0.9 mm diameter, World Precision Instruments, Berlin, Germany) positioned on the right side of the brain above the cerebellum (AP −11.0, ML 3.0) and frontal cortex (AP +4.0, ML 3.0) served as reference and ground electrodes, respectively. Before the electrocorticogram recording, a headstage composed of a screw and dental acrylic was attached to the right prefrontal part of the skull to hold the head in place and allowing removal of the right ear bar.

### Electrocorticogram recording

Continuous electrocorticogram was first 10-fold amplified using the AI 405 amplifier (Molecular Devices Corporation, Union City, CA, USA), high-pass filtered at 0.1 Hz, 200-fold amplified, and low-pass filtered at 400 Hz (CyberAmp 380, Molecular Devices Corporation), and finally sampled with a 16-bit precision at 2 kHz (DigiData 1320A, Molecular Devices Corporation). The data were stored on a computer hard disk using Axoscope 9.0 data acquisition software (Molecular Devices Corporation). The data analyses were performed offline using Vision Analyzer (Brain Products, Gilching, Germany), Matlab 7.5 (MathWorks Inc., Natick, MA, USA) and SPSS for Windows (SPSS Inc., Chicago, IL, USA).

### Stimulation

Sinusoidal tones of 50 ms in duration, including 5-ms rise and fall times, were used as stimuli. The tones were created using the Adobe Audition software (Adobe Systems Incorporated, CA, USA), and played from a PC via an active loudspeaker system (Studiopro 3, M-audio, Irwindale, CA, USA). The stimulation was presented with the passive part of the loudspeaker system directed towards the right ear of the animal at a distance of 20 cm. In all conditions, the sound pressure level for each tone was 70 dB, as measured with a sound level meter (type 2235, Bruel & Kjaer, Nærum Denmark) with C-weighting (optimized for 40–100 dB measurement) in the location where the animal's right pinna was during the recording.

For animal group 1, two stimulus conditions were presented. In the oddball condition, two infrequent deviants (probability of 0.0625 for each deviant type), one of 3800 Hz (‘deviant-3800 Hz’) and the other of 4200 Hz (‘deviant-4200 Hz’) were interspersed with frequently occurring (probability of 0.8750) standards of 4000 Hz (‘standard-4000 Hz’). We selected such a small difference (200 Hz, equals to 5%) between the standard and the deviant sounds in order to reveal the possible attenuation of the mismatch response from shorter to longer ISIs. The frequencies of the sounds were well above rats' hearing threshold [32]. The tones were delivered in a pseudorandom fashion with the restriction that consecutive deviants were separated by at least two standards. In the oddball condition two separate stimulus blocks were presented with inter-stimulus intervals of 375 ms and 600 ms (ISI, offset to onset).

The equiprobable condition comprised of 16 tones with frequencies ranging from 3300 Hz to 4800 Hz in 100-Hz steps (probability 0.0625 for each). The tones were presented with an offset to onset ISI of 375 ms. The range of tones applied also included those used as deviants in the oddball condition. We term these 3800 Hz and 4200 Hz tones presented in this condition ‘control-3800 Hz’ and ’control-4200 Hz’, respectively. Thus, these two tones had the same probability of occurrence as when they were used as deviants in the oddball condition.

In each block of the oddball condition (oddball-375 ms, oddball-600 ms), and in the block of the equiprobable condition (equiprobable), 1600 stimuli were presented. The order of the three stimulus blocks was counterbalanced across the animals, with pauses of 3–5 minutes between consecutive blocks.

For animal group 2, only two oddball stimulus blocks, with ISIs of 600 ms and 1000 ms, were applied. In addition, the difference in frequency between the standard and deviant stimuli was larger than that for the animal group 1, the deviants being 3500 Hz and 4500 Hz in frequency with the standard retained at 4000 Hz (500 Hz, equals to 12.5% difference). Otherwise the stimulation was carried out similarly as in group 1.

### Data analysis

The data were off-line filtered at 0.1–30 Hz (24 dB/octave). Sweeps from 50 ms before to 350 ms after stimulus onset were averaged for the oddball condition for each of the types of deviants and standards that immediately preceded these deviants. For the equiprobable condition, a similar procedure was applied for each type of control stimuli. The averaged waveforms were baseline-corrected against the mean of their 50-ms pre-stimulus period.

**Figure 1:**
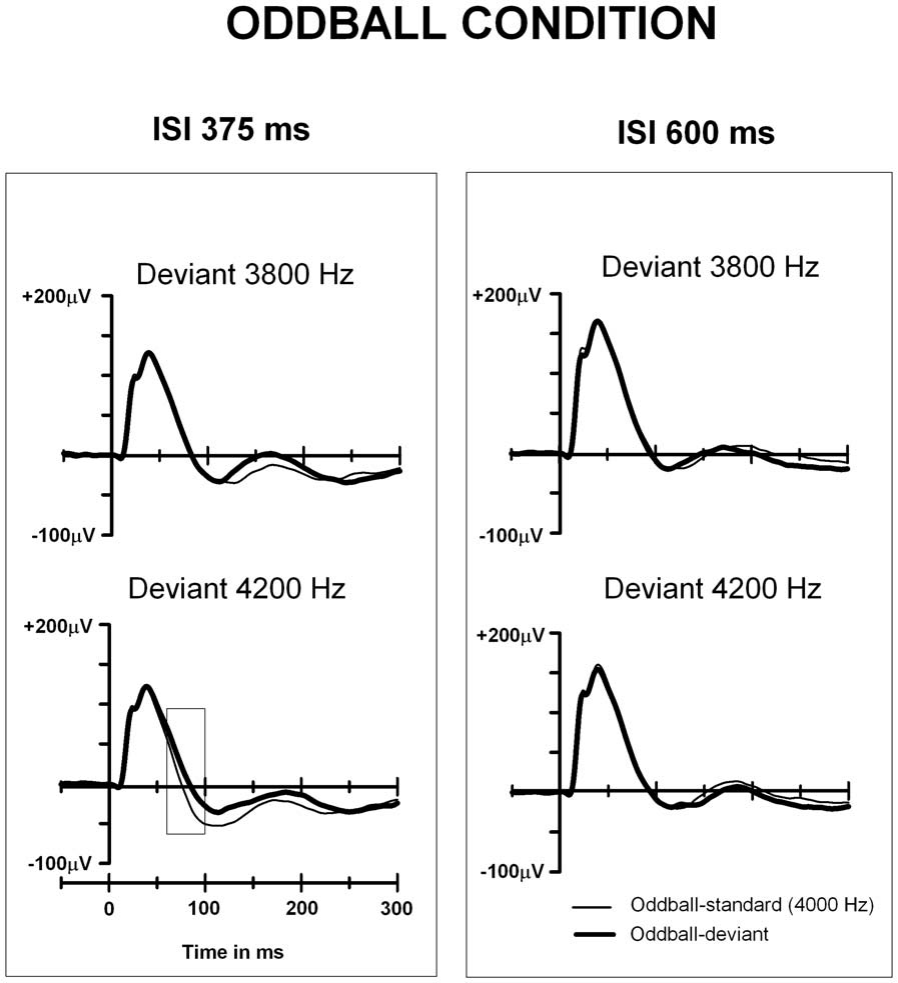
Local field potentials for the short (375 ms) and long (600 ms) ISIs in the oddball condition in animal group 1. Grand-averaged epidural potentials to standards and deviants elicited in the Oddball-375 ms and Oddball-600 ms conditions. The rectangle indicates the latency in which the significant difference between responses was found (60–100 ms after stimulus onset). The y-axis indicates the stimulus onset.

**Figure 2:**
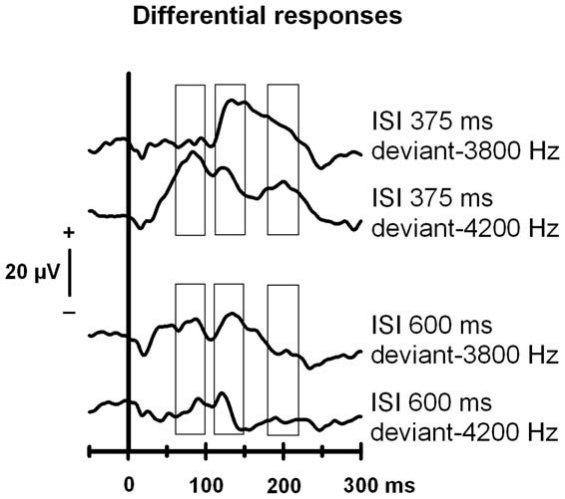
Difference waves for the oddball responses in animal group 1. Difference waves (deviant minus standard) in Oddball-375 ms and Oddball-600 ms conditions separately for both deviant types (deviant-3800 Hz and deviant-4200 Hz). The rectangles indicate the latency windows which have been applied in the data analysis (60–100 ms, 110–150 ms and 180–220 ms after stimulus onset).

Three different analysis windows were applied based on visual inspection of the peaks of the deviant-standard amplitude differences (Figures 1 and 2): 60–100 ms, 110–150 ms and 180–220 ms from stimulus onset. Mean local field potential values within these analysis windows were submitted to repeated measures analysis of variance (ANOVA).

The memory-comparison versus refractoriness hypotheses were tested by applying five levels for the factor Stimulus type for the conditions with an ISI of 375 ms, (standard-4000 Hz, deviant-3800 Hz, deviant-4200 Hz, control-3800 Hz, control-4200 Hz) applied in animal group1. Then separate ANOVAs with three levels of Stimulus type separately for the two animal groups and data from different ISI conditions were applied (standard-4000 Hz, deviant-3800 Hz, deviant-4200 Hz for the animal group 1 and standard-4000 Hz, deviant-3500 Hz, deviant-4500 Hz for the animal group 2).

Huynh-Feldt-adjusted degrees of freedom were used whenever the sphericity assumption was violated. Post-hoc analyses were carried out using two-tailed paired samples *t*-tests.

## Results

**Figure 3:**
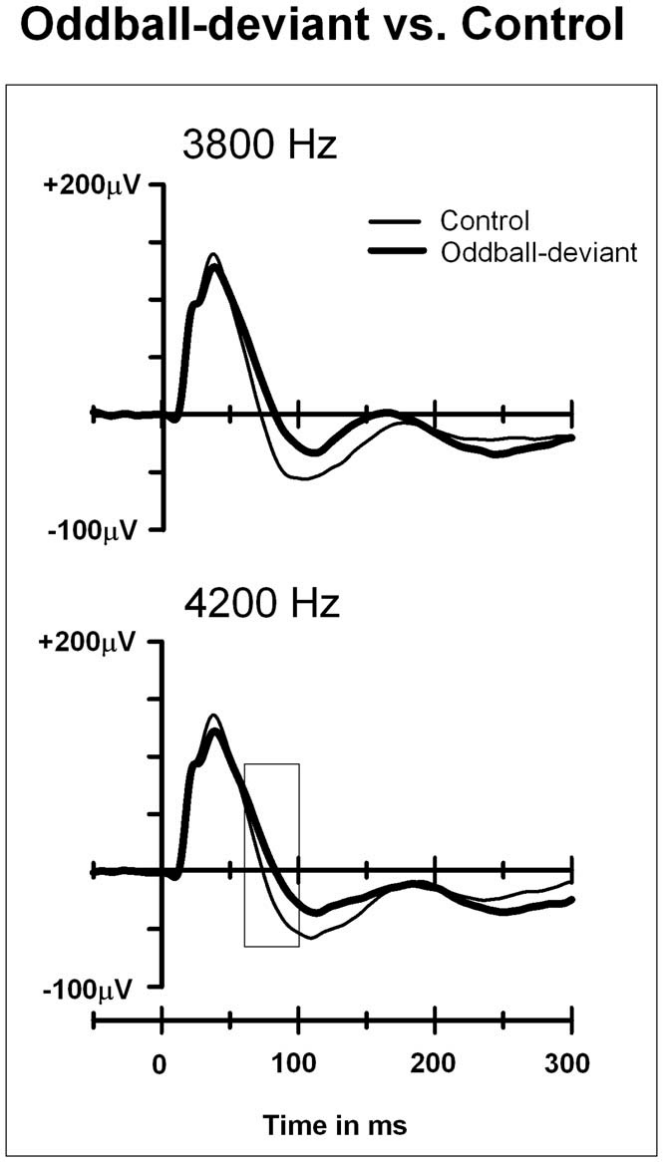
Local field potentials to the deviants in the oddball condition and to the control stimuli in the equiprobable condition in animal group 1. In both conditions the ISI was 375 ms. The rectangle indicates the latency in which the significant difference between responses was found (60–100 ms after stimulus onset). The y-axis indicates the stimulus onset.

**Figure 4:**
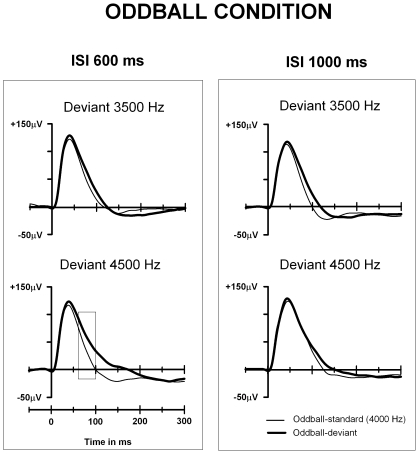
Local field potentials for the short (600 ms) and long (1000 ms) ISIs in the oddball condition in animal group 2. Grand-averaged epidural potentials to standards and deviants elicited in the Oddball-600 ms and Oddball-1000 ms conditions. The rectangle indicates the latency in which the significant difference between responses was found (60–100 ms after stimulus onset). The y-axis indicates the stimulus onset.

All types of stimuli evoked a prominent response of positive polarity peaking at ∼35 ms after stimulus onset (Figures 1, 3 and 4). This positive peak was followed by a smaller negative peak around 100–150 ms from stimulus onset. Visual inspection revealed a larger positive response to the higher frequency deviants (deviant-4200 Hz and deviant-4500 Hz) in comparison to the standards at approximately 60 ms to 200 ms post-stimulus in the shorter ISI conditions in the both animal groups (375-ms ISI in group 1, 600-ms ISI in group 2; Figures 1 and 4, respectively).

In the 60–100 ms analysis window for the 375-ms ISI condition (animal group 1), a significant effect of Stimulus type on the response amplitudes was found (standard-4000 Hz, deviant-3800 Hz, deviant-4200 Hz, control-3800 Hz, control-4200 Hz), F(4,76) = 3.1, p = 0.049. Further *t*-tests revealed a significantly higher response amplitude to deviant-4200 Hz than to standard-4000 Hz, t(19) = 2.7, p = 0.015, but no significant difference was observed between the responses to standard-4000 Hz and deviant-3800 Hz (Figure 1). Most importantly, the responses to deviant-4200 Hz were significantly higher in amplitude than those to the control-4200 Hz (the same tone presented in the equiprobable condition), t(19) = 2.6, p = 0.017. No significant difference was found between the responses to deviant-3800 Hz and control-3800 Hz (Figure 3). The mean amplitude was 14.7 µV for deviant-4200 Hz, -7.6 µV for the standard-4000 Hz preceding deviant-4200 Hz, and -27.2 µV for control-4200 Hz.

When the longer ISIs (600 ms) with deviant-3800 Hz and deviant-4200 Hz were used (animal group 1), no differences between the responses to the different stimuli were found. In addition, no differences were found when later time windows (110–150 ms and 180–220 ms after stimulus onset) were analyzed.

However, when the larger (12.5%) frequency separation between the standards and deviants were applied (animal group 2), differences between the stimulus types were found also with 600 ms ISI. Responses to standard and deviant tones in Oddball-600-ms and Oddball-1000-ms conditions for the animal group 2 are shown in Figure 4. In the analysis window of 60–100 ms after stimulus onset, there was a significant effect of Stimulus type (standard-4000 Hz, deviant-3500 Hz, deviant-4500 Hz), F(2,24) = 5.5, p = 0.011. Subsequent *t* tests revealed that this effect was due to a significantly more positive response to the deviant-4500 Hz tones compared to standard-4000 Hz tones, t(12) = 2.7, p = 0.019, but a lack of such differential response between deviant-3500 Hz and standard-4000 Hz (Figure 4). The mean amplitude was 62.6 µV for the deviant-4500 Hz and 37.2 µV for the standard-4000 Hz tone preceding it.

When a longer ISI (1000 ms) was used, no differences between responses to the different stimuli were found. In addition, no differences were found when later time windows (110–150 ms and 180–220 ms after stimulus onset) were analyzed.

## Discussion

Brain electrical responses were recorded epidurally in urethane-anesthetized rats above the auditory cortex (A1) during auditory oddball experiments. In both groups of animals, a differential response of positive polarity (deviant minus standard, i.e. a mismatch response) was observed at 60–100 ms from stimulus onset for melodically ascending (deviants of 4200 Hz or 4500 Hz) but not for descending (deviants of 3800 Hz or 3500 Hz) changes in a series of repeating standard stimuli of 4000 Hz. The mismatch response disappeared when the ISIs were prolonged from 375 ms to 600 ms (+5% change in frequency) in group 1 and also when the ISIs were prolonged from 600 ms to 1000 ms (+12.5% change in frequency) in group 2.

The attenuation of the mismatch response from shorter to longer ISIs probably indicates that the neural representation of standards provided a template for the deviant detection of a +5% deviation from the standard frequency and was maintained for at least 375 ms. This representation is likely to depend on the magnitude of deviance and fades rapidly over longer periods, but after 600 ms, it still allowed the detection of a +12.5% deviation from the standard frequency, most probably because a more robust representation of the deviants. The 5% difference in frequency applied between the standards and deviants was close to the behaviorally assessed Weber ratios of frequency difference limen in awake rats (from 3.7 to 7.3%, [33]). Thus, the memory trace had to be highly accurate in representing the standard frequency for at least 375 ms.

In the present study mismatch responses were observed only for melodically ascending deviants. A similar asymmetry in change detection favoring the ascending over descending frequency deviations has previously been found in humans using both neurophysiological [34], [35] and behavioral [36] measures. Together these and the present finding suggest a novel phylogenetic continuum in central auditory processing, one that has possibly evolved across animal species in response to shared environmental demands on the auditory system.

An important aim of the present study was to test whether the memory trace formed by the standards is needed to elicit the mismatch response. To this end, we applied a control condition where no repetitive standards were presented, i.e. the equiprobable condition [11], and found that the response to the control stimulus was lower in amplitude compared to the response to the deviant stimulus recorded in the oddball sequence. This suggests that neuronal refractoriness alone cannot account for the difference in potentials we observed for the rare oddball deviants. Unlike the deviant-alone condition used in previous studies in anesthetized rats to test the refractoriness hypothesis [23], [26]–[28], the equiprobable condition applied here preserved the same overall presentation rate of the stimuli as in the oddball condition. For this reason the equiprobable condition is a more valid control for possible refractoriness effects than the deviant-alone condition.

One may argue that the responses to the 3800 Hz and 4200 Hz tones may be smaller in amplitude in the control than in the oddball condition because of the smaller frequency range between tones in the control condition (steps of 100 Hz in equiprobable condition vs. 200 Hz in oddball condition). However, it is more likely that the opposite is true, namely, because the tones in the equiprobable condition are presented randomly from a wider frequency range than in the oddball condition. Thus, the frequency separation between control stimulus and previous (or nearby) tones can be much larger than the standard-deviant frequency separation in oddball condition; hence frequency-specific refractoriness is less likely to occur (see also [11]). Please note also, that although no equiprobable condition was applied with the 600-ms ISI (only for the 375-ms ISI), refractoriness effects are unlikely to account for the mismatch response observed using the longer ISI either, as the oddball-deviant vs. equiprobable control stimulus comparison ruled it out for the shorter (375-ms) ISI condition which is more prone to possible refractoriness effects.

Mismatch responses have been previously observed in animals with various anaesthetic agents, including urethane [16], [21], [23], ketamine [20], [29], and pentobarbital [18], [27]. On the other hand, mismatch responses in intracortical field potentials have not been observed in awake rats, even for frequency changes that have clearly been above the discrimination threshold, when 800-ms silent ISIs have been used [25]. Thus, it seems likely that urethane-induced anaesthesia may leave the memory and change detection functions of the rat auditory system unaffected. In humans, propofol anaesthesia has led to inconsistent findings of MMN during deep sedation (e.g. [37], [38]). Further systematic enquiries on the effects of the type and the level of anaesthesia on MMN as well as on its animal analogues would provide invaluable information on the neuropharmacology of the core perceptual mechanisms.

While the epidural local field potential recording applied here reflects synchronized post-synaptic activity of hundreds or thousands of neurons, intracranial single cell recordings provide a different level of analysis to the auditory change detection. Neuronal stimulus-specific adaptation (SSA) to a repeated sound, which does not fully generalize to the other sounds, has been suggested as a single-cell correlate of scalp-recorded MMN [39]. SSA to repeated sound frequency was initially reported in cat auditory cortex [40] and relatively weak levels of SSA was also found in the auditory cortex of awake rats [25]. In addition, some subcortical structures in rats contribute significantly to the elicitation of SSA (inferior colliculus: [41]; medial geniculate body: [42]; for a relatively weak levels of SSA in mice, see also [43]). Since some of these studies have been carried out in the same preparation as the one used in the present study, i.e. in urethane anesthetized rats, a closer inspection of their results is fruitful. In the medial geniculate body of the thalamus [42], SSA was best observed when the non-stimulated ISI was 150 ms or 400 ms while only few neurons responded in a stimulus-specific manner when ISI was 2000 ms. This finding is in agreement with the present results showing degrading of the mismatch response from shorter to longer ISIs. In the inferior colliculus [41], the difference between responses to the deviant and standard stimulus was positively correlated with the amount of frequency separation between the stimulus types. This is well in line with our results showing that the larger frequency separation allowed elicitation of the mismatch response with the same ISI (600 ms) that was too long when the smaller separation was applied. It is noteworthy that no studies of SSA have applied the equiprobable control condition (or deviant-alone condition either). This control condition would allow the testing of whether SSA could serve as a neural basis for the memory-based change detection mechanism. In bridging this gap between MMN and SSA, local field potentials recorded simultaneously with single-cell responses (see ref. [25]) would provide a valuable method.

In conclusion, we observed mismatch responses in urethane-anesthetized rats to rare auditory changes with a set of characteristics similar to those of MMN in humans. These characteristics include higher sensitivity to ascending than descending frequency changes [34], dependence on the inter-stimulus interval (e.g. [5]), and a memory-based comparison process as an underlying mechanism (e.g. [11], [12]).
